# Monitoring supply networks from mobile phone data for estimating the systemic risk of an economy

**DOI:** 10.1038/s41598-022-13104-5

**Published:** 2022-08-03

**Authors:** Tobias Reisch, Georg Heiler, Christian Diem, Peter Klimek, Stefan Thurner

**Affiliations:** 1 Section for Science of Complex Systems, Center for Medical Statistics, Informatics and Intelligent Systems, Medical University of Vienna, 1090 Vienna, Austria; 2 Complexity Science Hub Vienna, 1080 Vienna, Austria; 3 Institute of Information Systems Engineering, TU Wien, 1040 Vienna, Austria; 4 Institute for Finance, Banking and Insurance, Vienna University of Economics and Business, 1020 Vienna, Austria; 5 Santa Fe Institute, Santa Fe, NM 85701 USA

**Keywords:** Complex networks, Applied physics

## Abstract

Remarkably little is known about the structure, formation, and dynamics of supply- and production networks that form one foundation of society. Neither the resilience of these networks is known, nor do we have ways to systematically monitor their ongoing change. Systemic risk contributions of individual companies were hitherto not quantifiable since data on supply networks on the firm-level do not exist with the exception of a very few countries. Here we use telecommunication meta data to reconstruct nationwide firm-level supply networks in almost real-time. We find the probability of observing a supply-link, given the existence of a strong communication-link between two companies, to be about 90%. The so reconstructed supply networks allow us to reliably quantify the systemic risk of individual companies and thus obtain an estimate for a country’s economic resilience. We identify about 65 companies, from a broad range of company sizes and from 22 different industry sectors, that could potentially cause massive damages. The method can be used for objectively monitoring change in production processes which might become essential during the green transition.

## Introduction

Bilateral interactions between the agents in an economy lead to networks that dominate practically all aspects of the economy, ranging from networks of production^1,2^, finance^3^, distribution^4^, consumption^5^, and recycling^6^. Networks are not only the basis of an efficient functioning of the economy, they are also the source of some of its implied risks and, in particular, systemic risk, or the risk that a large fraction of networks stop to function and do no-longer fulfil their function. Remarkably, the understanding of the economy in terms of its underlying networks has not arrived at mainstream economics^7^. This work focuses on two particular types of networks that are closely related. Supply networks describe the flow of goods between economic actors, typically firms. Production networks are a combination of supply networks and a description of the production processes happening at the nodes, the node production function.

Since about two decades systemic risk has been associated with network structures and ways to quantify it are nowadays available.The main idea behind the quantification of systemic risk is to estimate the economic or financial consequences of a defaulting node or link in a given network on the entire system. The fraction of the total system affected is typically associated with the systemic risk of a node or link. Knowing the systemic risk contributions of agents offers a way to quantify the resilience and robustness of a system. The first networks available to research were financial networks such as networks of inter-bank claims and liabilities^3^, or of overnight money markets^8^. Systemic risk in these networks was first quantified with network measures like betweenness centrality^9^, which were later improved by explicitly incorporating economic default mechanisms and the associated accounting procedures^10,11^. Further extensions involved multilayer networks^12,13^, overlapping portfolios^14,15^, in the context of financial networks, as well as some applications in the real economy^16^, and lately, also in production networks^17,18^.

Systemic risk in mainstream economics has often been discussed not on the basis of networks^19,20^, but on financial time series data that obviously can’t account correctly for cascading processes. It is exactly the cascading that leads to extraordinary large effects that are often associated with the fat tailed distributions of losses^21^. The default of Lehman Brothers in 2008^22,23^, the 2008-2010 global food crisis^24^ and, more recently, world wide supply chain disruptions due to the COVID-19 pandemic^25,26^ are only a few examples of severe events in financial markets, basic provision, or production networks, where cascading plays an essential role.

A network-based quantification of systemic risk makes it possible to identify the weak points in these systems and consecutively allows one to design mitigation strategies, for example an adaptive systemic risk tax to reduce the systemic risk in a banking system^27,28^ or the computation of optimal networks that carry a minimum of systemic risk^14,29^. However, the computation of systemic risk requires the detailed understanding of the structure and dynamics of the underlying networks, which hitherto posed a major challenge^30^.

**Figure 1:**
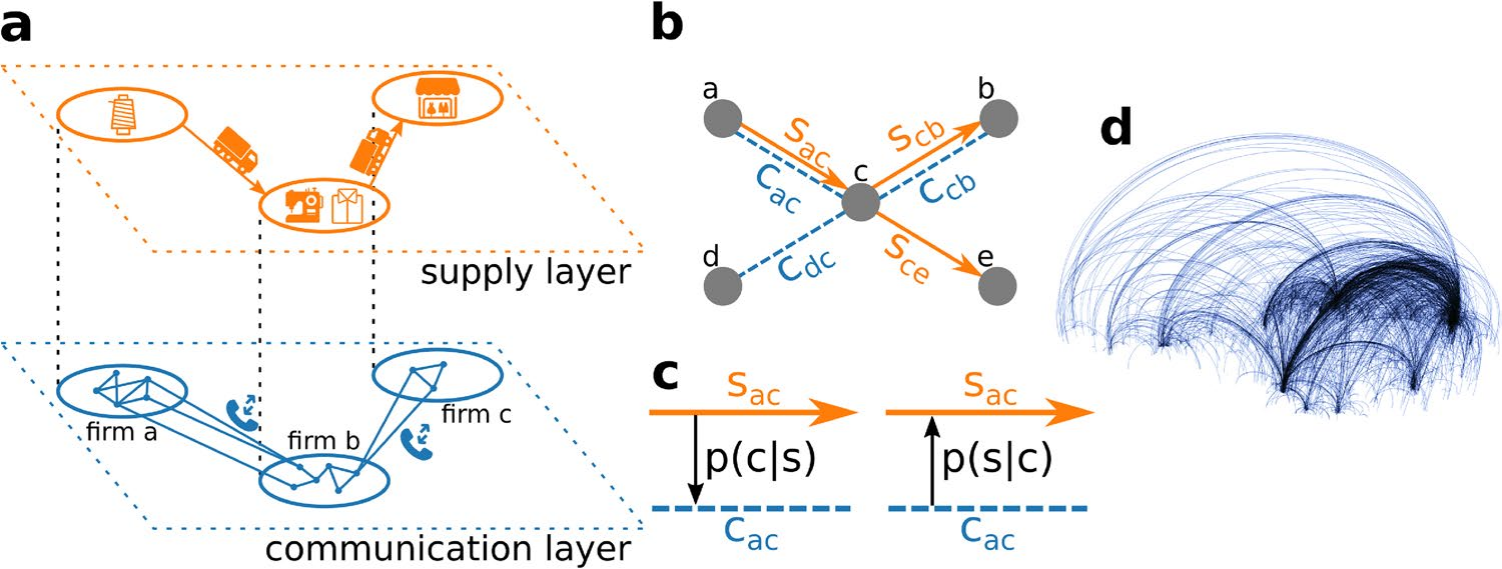
(**a**) Schematic view of the inter-firm multilayer network with a communication layer (blue) of phone calls between groups of devices that are associated to firms and the supply layer that captures the actual flow of goods (orange). (**b**) Section of the multilayer network where communication links, $c_{ij}$, exist if at least one phone call between firms *i* and *j* takes place and supply links, $s_{ij}$, exist if goods flow from *i* to *j*. (**c**) Conditional probabilities between supply links and communication links are defined as the probabilities to find a supply link, conditional on a communication link being present, *p*(*s*|*c*) and vice versa for *p*(*c*|*s*). (**d**) The inter-firm communication network as provided by a mobile phone company. Arcs link firms that have an average call duration of more than 150 s/d. Firms are slightly dislocated randomly, enough to ensure the anonymity of companies.

This is particularly true for systemic risk in production networks. Only for very few countries buyer-supplier relations are known on a granular level of individual companies from which the supply-chain networks can be constructed. For Hungary value-added tax (VAT) data exists that specifies which company pays VAT to another. From this the *exact* national supply-chain network has been reconstructed^31^, containing more than 89,000 companies and 235,000 business relations (links). Using estimations for production functions for these companies makes it possible to obtain the national production network. Using this as an input, firm level systemic risk for all individual companies were computed by using an appropriately designed SR measure, the Economic Systemic Risk Index (ESRI)^18^. It is a network-based measure to estimate the fraction of the total production output (goods and services) of the economy that is affected by a firm’s (short-term) failure.

However, the Hungarian data is an exception. Granular and exhaustive datasets on the supply network of an entire nation are notoriously hard to obtain. Data exists only for a handful of countries, Japan^1^, Belgium^2^, Brasil^32^, and Hungary^31^. Customer-supplier relations are inferred either from surveys and business intelligence^1^, payment system data^32^, or VAT data^2,31^. Survey data is typically very costly to collect and suffers from being outdated, highly incomplete, unweighted, and hard to verify^30^; on the other hand, payment system and tax data –in countries where it is collected– is sensitive and access is highly restrictive.

In this work we propose an alternative approach to reconstruct the supply-chain network by using the multilayer network structure of firm-to-firm relations. The coordination of a customer-supplier relation, such as ordering, negotiating prices, or organizing shipping, requires communication between firms and has been studied intensively^33,34^. We thus hypothesize that companies that communicate with each other also entertain customer-supplier relations. Therefore, we focus on two network layers, the flow of goods and services that constitute supply relations and the mobile phone communication between companies. Figure 1a schematically depicts the two-layer network. The communication layer (blue) shows the mobile devices belonging to one firm, calling devices in other firms. The supply layer (orange) represents the flow of intermediate products (or services) between firms. In Fig. 1b we show the same situation by showing a communication link $c_{ij}$ (blue) between firm *i* and *j* if they had at least one phone call within a certain time period and a supply link $s_{ij}$ (orange) if goods or services flow from firm *i* to *j*. Note that communication links are undirected, supply links are directed.

We expect the existence of strong link-correlations between the communication and supply layers. To quantify these link-correlations, we start from the multilayer network in Fig. 1b, and define the conditional probability, $p(s_{ij}|c_{ij})$, to find a supply link, $s_{ij}$, between firms *i* and *j* given that a communication link, $c_{ij}$, exists, and vice versa, the conditional probability, $p(c_{ij}|s_{ij})$, to observe a communication link given that a supply link exists, see Fig. 1c.

Albeit strong legal regulation, telecommunication data has been accessible to researchers for more than a decade. Mobile phone data in the form of call detail records (CDRs), that are collected by mobile phone operators for billing purposes, have been used to study communication networks and the behavior of millions of people^35^, leading to spectacular insights into the structure of human communication and organization^36,37^, human behavior in emergency situations^38^, the spread of infectious diseases^39,40^ and the principles of human mobility^41–43^. CDRs allow for population-wide coverage, granular resolution of interactions on the person level, and the possibility to be combined with information, such as age and gender. Even though possible, inter-firm or organisational networks have so far not been studied systematically with mobile phone data.

Through a cooperation with a large mobile phone provider, we have access to a dataset of CDRs in a medium-sized European country. This allows us to identify groups of phones that are associated with a company through anonymized billing information, for details, see Materials and Methods. The dataset contains additional information on the firm’s primary industry classification and balance sheet information. The cooperation partner is one of several large mobile phone providers that have approximately equal market share. The final dataset contains a five-digit number of companies. The Pearson correlation between firms per sector in our sample and all firms in the country is r = 0.84. This indicates that the sample is representative of the entire economy, see SI Fig. S1 and SI Text 1. In Fig. 1d we show the corresponding firm-to-firm communication network (FCN) as obtained from our data. Firm locations are shifted by random distances (on average 30 km) to ensure the anonymity of companies. Arcs in the figure represent communication links between firms. We find many short-range interactions within one city or economic region and few long-range interactions. We are intentionally vague with regards to information concerning the mobile phone provider, because we are contractually bound to ensure its anonymity, as well as to protect sensitive business information such as the exact market share in the business-to-business market.

Here we demonstrate that phone data can indeed be used to reasonably reconstruct supply networks that allow for a meaningful estimation of firm-level economic systemic risk of an economy. The method is an efficient alternative to survey, tax, or bank transactions estimates. It uniquely allows us to study supply networks and monitor economic systemic risk in real time and provides a nearly complete overview of a nation’s production network.

## Results

**Figure 2:**
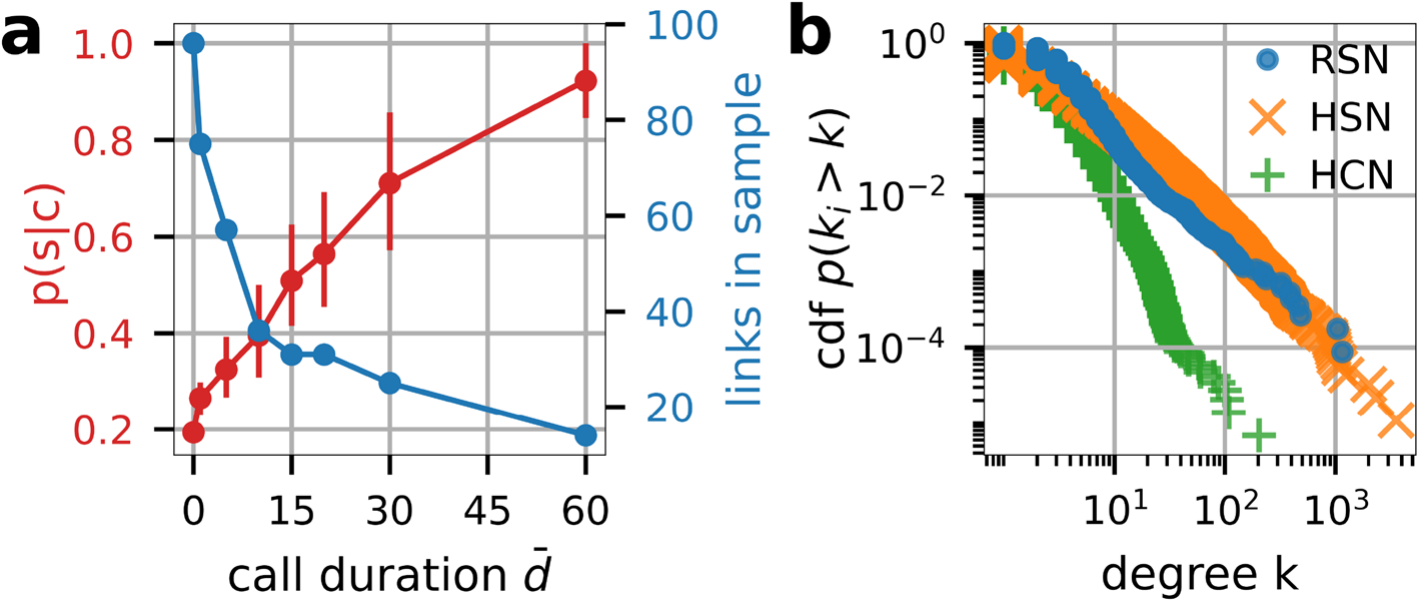
(**a**) Probability *p*(*s*|*c*) to find a supply link, $s_{ij}$, given that there exists a communication link, $c_{ij}$, between firms *i* and *j* for communication links exceeding a given call duration, ${\bar{d}}_{ij}$. Error bars denote the quartiles of a bootstrap simulation described in SI Text 2. (**b**) Cumulative distribution function $p(k_i>k)$ for the degree *k* of the reconstructed supply network, RSN, (blue dots), Hungarian supply network, HSN, (orange x’s) and human communication network, HCN, (green pluses). The degree distribution of the HSN is much more similar to the RSN than to the HCN.

### Conditional supply-link probability

We determine the conditional supply-link probability *p*(*s*|*c*) by comparing the firm communication network, shown in Fig.  1d, with ground-truth information on the real customer-supplier relations, obtained from a nation-wide survey among the members of a large business representation organization that represents firms across all sectors except agriculture, conducted in April 2020. In the online survey more than 100,000 companies and businesses were asked to share their ten most critical suppliers and customers, respectively. More than 5,900 firms declared at least one supplier or customer with a total of more than 17,000 customer-supplier relations reported. For details on the survey, see SI Text 2. We obtain the overall probability that a supply link exists between two companies, given that they had at least one conversation event in the observed time period of approximately 125 days, is $p(s|c) = 0.19$. For the conditional communication probability we get $p(c|s) = 0.27$. For comparison, the respective marginal probability from the firm communication network directly is $p(c)=0.002$. For the linking probability --using Hungarian data-- we get $p(s)=0.00005$. Both values are orders of magnitude smaller than the conditional probabilities, indicating highly significant link correlations between the supply and communication layers.

The conditional link probability increases with the intensity of the firm-firm communication. As a proxy for the latter we use the average daily call duration, ${\bar{d}}_{ij}$, in seconds per day. In Fig. 2a *p*(*s*|*c*) is shown conditional that ${\bar{d}}_{ij}$ is larger than a threshold ${\bar{d}}$ (red). The number of links used to calculate the overlap decreases as a function of the threshold ${\bar{d}}$ and is shown in blue. *p*(*s*|*c*) rises from 19% to values around 70% for ${\bar{d}}_{ij}>30$s/d and around 90% for 60s/d. The number of links reduces from 75 to 14 links as ${\bar{d}}$ increases. Note that errors do not increase, because a higher probability is associated with a smaller error. For details of the computation and errorbars, see SI Text 2.

For the supply network (*p*(*c*|*s*)) the best proxy for tie strength would be the amount of traded goods. However, this information is not available. Following the philosophy of “gravity models” in economics, we assume that large and small firms typically trade large and small volumes, respectively^44^. The gravity model implies that the link weight is proportional to the product of the firm’s sizes. Here, to stay consistent on the communication data, we proxy the firm size with the number of devices associated with a firm. Supplementary Fig. S3 shows *p*(*c*|*s*) for the networks thresholded by the number of devices per firm in red and the number of links in the underlying sample in blue. We find an increase from 27% to around 60% for the network of firms with 4 or more devices. For thresholds larger than 4, the curve levels off and stabilizes around 70% for thresholds of 6 or more devices. The number of links drops as in Fig. 2a, but again, the error-bars are still sufficiently small.

### Reconstructing the supply network

To obtain an estimate of the supply network, based on the communication network, c, we assume all communication links, $c_{ij}$, with a call duration of more than 30 seconds per day,  ${\bar{d}}_{ij}=30$s/d, to signal a supply relation, $s_{ij}$. With this threshold we aim to balance the loss of information due to ignored supply links and increasing link correlations due to the thresholds. This particular threshold is the result of a minimization of the Kullback-Leibler divergence for degree distributions of the Hungarian supply network (HSN) and thresholded FCNs, described in SI Text 3. We arrive at an unweighted and undirected reconstructed supply network (RSN). To get an estimate for the link directions (firm *i* supplies *j,* or vice versa), we use classical input-output tables of the national statistical office. They contain information on the volume of trade between economic sectors in the economy. An element of the input-output table, $G_{ab}$, describes the flow of goods (in Euro) from sector *a* to sector *b*. We denote the number of links (firm-firm supply relations) from sector *a* to sector *b* by $L_{ab}$ and assume that the ratio of links from one sector to the other is proportional to the ratio of goods flowing between these sectors, ${L_{ab}}/{L_{ba}} \approx {G_{ab}}/{G_{ba}}$. For example, the flow between the agricultural sector (*a*) and the food industry (*f*) is $G_{af} \approx 3,400 \mathrm {m}$€, while the food industry sold goods for $G_{fa} \approx 450 \mathrm {m}$€ to the agricultural sector. We now assume that it is${3,400}/{450} \approx 7.6$ times more likely that a supply link points from a firm *a* to one in *f*. We now consider every link from firm *i* in sector *a* to firm *j* in sector *b* in the RSN and assign it a direction according to the probability

1 $$\begin{aligned} p(i \rightarrow j) = \frac{G_{ab}}{G_{ab}+G_{ba}} \, . \end{aligned}$$

Since we perform this assignment stochastically, we should think in ensembles of RSNs. Finally, we estimate a supply-link weight for every link in the RSN. We use the companies’ total assets, calculated from the balance sheets, as size information, $s_i$; it is obtained from a commercially available business intelligence database, see Materials and Methods. As before, in the spirit of “gravity models”^44^, we estimate the link weight between firms *i* and *j* proportional to the product of firm sizes, $W_{ij} = s_i s_j$. We will use only relative weights in the following.

### Comparing network topologies of supply-chains, firm-firm communication, and human communication

It is enlightening to compare the network topology of the so-obtained RSN (blue in Fig. 2b) with the topologies of the Hungarian supply network (HSN) (orange) (for which the exact topology is known^18^) and the private communication network between individual people (green) (i.e. not between companies). Figure 2b shows the degree distribution of the RSN (blue) in comparison to the exact HSN derived from VAT data^31^. Both networks are similar and fat tailed, in contrast to the human communication network (HCN) that was obtained from the mobile phone data set. The RSN has an average degree of $\langle k^{RSN} \rangle = 4.79$. Its degree distribution has a maximum at $k^{RSN}=2$ and its fat tail can be approximated by a power law exponent $\alpha _k^{RSN} = 2.18(12)$ for $k^{RSN}>30$. The HSN does not show an increase for small *k* but also exhibits a fat tail with $\alpha _k^{HSN} = 2.40(3)$, for $k^{HSN}>30$. The average degree is $\langle k^{HSN} \rangle = 2.1$. For the HCN we find an average degree of $\langle k^{HCN} \rangle = 4.75$. There, the decrease of *p*(*k*) for high values is stronger, with an exponent of $\alpha _k^{HCN} = 4.89(26)$ for $k^{HCN}>20$. For a more detailed comparison of network characteristics, including the clustering coefficient and nearest neighbor degrees, see SI Text 4, SI Fig. S5 and SI Tab. S1.

**Figure 3:**
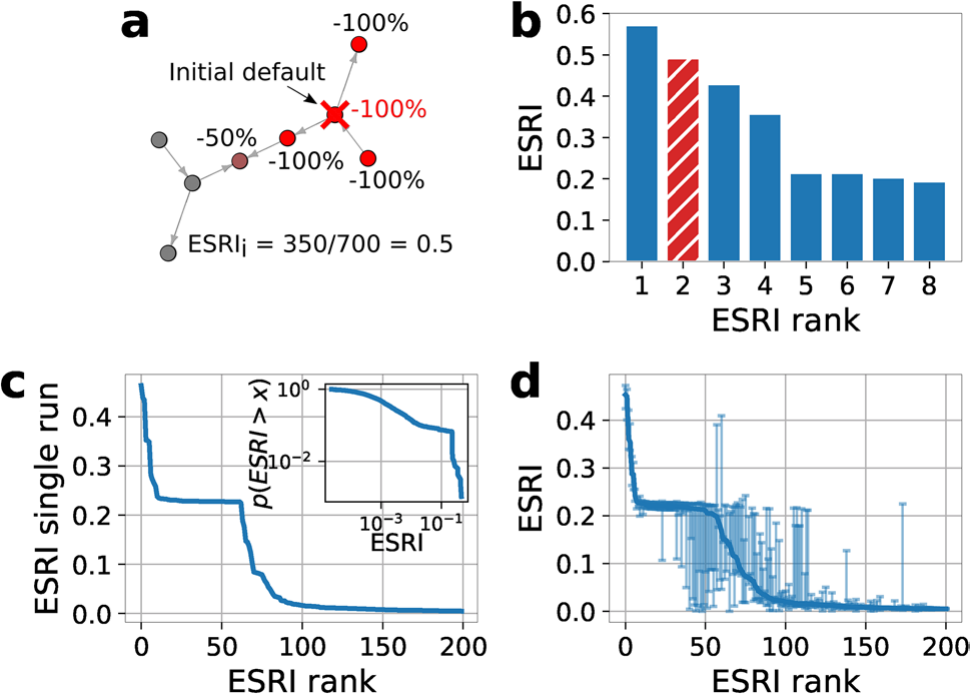
Economic systemic risk in production networks. (**a**) Toy example of a production network with eight firms of the same sector and size. After the default of an initial node (red X) its customers (suppliers) have to reduce their production level according to the share of inputs (supply) they lost. This logic is iterated until a stable configuration is reached, the relative share of economic activity (production) lost is the initial node’s ESRI. (**b**) The systemic risk profile for the toy network in panel (**a**), the initial node in panel (**a**) is highlighted in red. The bars show the ESRI for all firms sorted according to ESRI, from highest to lowest. (**c**) Rank ordered systemic risk profile (most risky to the left) for one realization of the RSN. There are 65 high systemic risk firms forming the visible plateau, and a rapid decrease in ESRI for higher rank firms. The maximum ESRI is 0.47, the majority of the plateau has an ESRI of around 0.21. The inset shows the counter cumulative distribution function $p(ESRI>x)$ in double logarithmic scale, excluding values of $ESRI<10^{-5}$. (**d**) ESRI profile for 100 realizations of the RSN. For every firm we show its median ESRI of 100 RSNs, the firms are ranked according to the median ESRI (solid blue line), the error bars show the 25% and 75% percentile. For the median ESRI we find a slightly smaller core of around 50 high systemic risk firms with the majority of the risky firms having a median ESRI of around 0.22 and a maximum median ESRI of 0.45. The error bars are small for high and low systemic risk firms, but large for firms in-between, suggesting that their ESRI strongly depends on the direction of one or a few links.

### Economic systemic risk

With a reasonable reconstruction of the supply network, RSN, we turn to the quantification of economic systemic risk in the national production network. For this we use the economic systemic risk index (ESRI) as developed in^18^. The underlying principle of the ESRI algorithm is sketched in Fig. 3a, in an example with seven firms of equal size within the same industrial sector. The ESRI of firm *i*, assumes that if firm *i* (red cross) cannot operate for some time (e.g. defaults) it neither supplies nor demands inputs. The customers and suppliers of *i* then reduce their production accordingly, causing a successive reduction of production in their customers and suppliers. This recursive reduction converges to a state where all firms have reduced their level of production in response to *i*’s default. Figure 3a shows the relative reduction for every firm. The fraction of total economic activity lost is the $ESRI_i$ of firm *i*. We use the ESRI with a heuristically calibrated generalized Leontief production function (SI Eq. (9)), which captures the major differences between firms with physical production functions (e.g. agriculture, manufacturing, etc., i.e. NACE A01-F43) and firms producing services (NACE G45-U99). For the first group, physical inputs (i.e. products supplied by firms from NACE A01-F43) are essential to their production process and, consequently, their lacking causes major disruptions in firm's production in a non-linear fashion. In economic terms, these are Leontief inputs. However, service inputs such as consulting services, travel agency services etc. are not essential for the physical production and disrupt production only in a linear way. For firms producing services we assume that all inputs disrupt their production in a linear fashion. For a detailed explanation of the use of generalized Leontief production functions in the ESRI definition, we refer to SI Text 5 and the “Generalized Leontief” scenario in^18^.

We compute the ESRI for every firm in the network and plot their values according to their rank, from highest ESRI to lowest, in Fig. 3b. This is called the *systemic risk profile* of the production network. The ESRI for the defaulting firm in panel a is highlighted as the red bar. Performing the same steps for all firms in one realization of the RSN yields the systemic risk profile shown in Fig. 3c, where we show the 200 riskiest firms. The profile shows similar characteristics to what has been reported for the exact production network of Hungary^18^, namely, a plateau containing the 65 most risky firms, which all, except for a few extremely risky firms, have a similar risk of around $\mathrm {ESRI} \approx 0.21$, followed by a sharp decline for firms that are not part of the plateau. The inset in Fig. 3c shows the cumulative distribution (CDF) $p(\mathrm {ESRI} > x)$ of the ESRI in log-log scale.

To take the stochastic nature of the RSN into account we repeat the ESRI calculation. We consider five realizations of the RSN to calculate their mean ESRI. Subsequently, due to computational challenges, we focus on the 1000 most risky firms only, after ranking them according to their mean ESRI. For those we repeat the ESRI calculation 100 times. For each node we get a distribution of ESRI values. Figure 3d shows the median ESRI for every firm as a solid line; the 25% and 75% quantiles are indicated by the errorbars. An alternative way to investigate the ESRI profile of the RSN is to plot the maximal systemic risk of every node. This method yields similar results and is shown in SI Fig. S6 in SI Text 6. The median ESRI per node profile in Fig. 3d shows the same characteristics as the single run in Fig. 3c, a plateau of high-risk firms and a rapid decline of ESRI outside of the plateau. In contrast to the single run ESRI profile, the plateau consists of only around 50 firms. The spread of the ESRI distributions for individual nodes is small for high- as well as low-risk nodes, indicating that the results are remarkably stable and robust. For the intermediate risk firms error-bars become large, indicating that their ESRI depends on the direction of one or few links. It is a well-known feature of systemic risk and the ESRI that single links or link directions can have a large influence^11,18^. Ref.^18^ explains that some nodes “inherit” systemic risk by being a crucial supplier to a firm that is inherently risky due to e.g. its size. Therefore flipping a link-direction can turn a node from a crucial supplier of a central firm to a buyer of that firm, which strongly reduces its inherited systemic risk.

**Figure 4:**
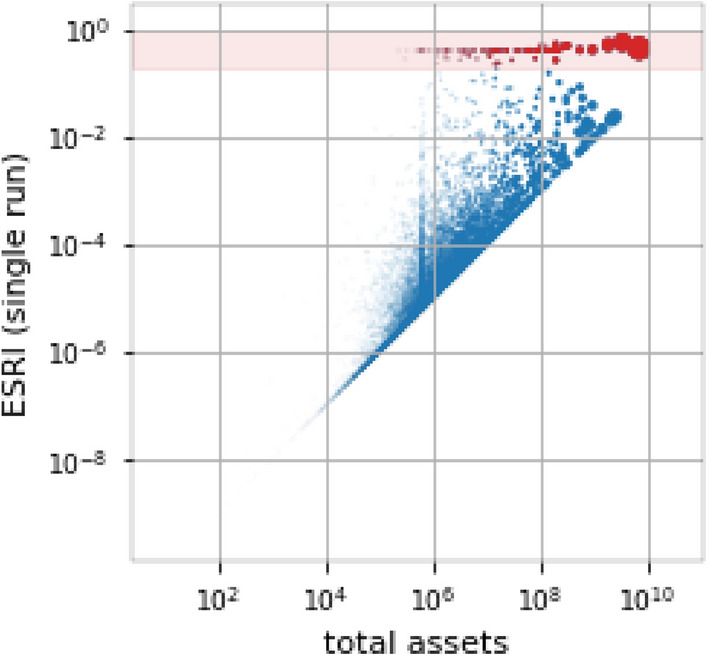
Economic systemic risk vs. firm size measured as total assets in log-log scale. Marker size represents total assets; companies in the “plateau” of Fig. 3c are marked red and highlighted by red shading. For a given firm-size there is an obvious lower bound for the ESRI that corresponds to the firm-size (here no firm can loose less than all assets when it defaults). Although the correlation between size and ESRI is high, we find small and large firms in the “plateau”, suggesting that firm-size is not a good tool to identify high-systemic risk firms.

To understand which firms are in the plateau of Fig.  3c, in Fig. 4, we plot the firm-size, approximated by the firms’ total assets against the ESRI. It is evident that the high systemic risk plateau (highlighted in red) contains large and small firms, with their total assets spanning more than 4 orders of magnitude. Although firm size correlates well with ESRI (Spearman’s $\rho =0.87$), it is not a good predictor for systemic risk, since for a given firm-size the ESRI can vary by several orders of magnitude. A similar situation is described in^18^.

The 65 firms found in the high systemic risk plateau mainly belong to the manufacturing sector (NACE lvl. 1 category C, 77%), followed by companies in the electricity, gas stream and air conditioning supply (D, 8%) and financial and insurance activities (K, 6%) sectors. The full composition comprises 22 NACE lvl. 2 sectors and is listed in SI Tab. S2. In contrast to the exact Hungarian production network^18^, several companies from non-manufacturing sectors (NACE $\ge$ 45) are found in the plateau. This is somewhat unexpected since they are associated with linear production functions (see SI Text 5), which causes their shock spreading behavior to be less extreme than for Leontief producers.

### Robustness of results

Our study is subject to several limitations, in particular (i) the imperfect overlap of the two communication and supply-link layers, limiting the possible accuracy, (ii) the limited market coverage of the phone provider (resulting in limited agreement even if $p(s|c) = 1$), see SI Text 7, and (iii) errors originating from the network reconstruction uncertainties in the estimations of directions and weights.

To estimate the biases and errors introduced by these weaknesses, we perform several simulation studies. First, we generate a synthetic communication network based on the HSN and the probabilities to find a communication link, where a supply-link is present *p*(*c*|*s*), and where no supply-link is found $p(c|\lnot s)$. From this synthetic communication network we then take a sample of nodes according to an estimated market share *m* of the data provider and calculate the induced subgraph comprised by links only between the sampled nodes.

Finally, following the procedure used on the empirical data, we reconstruct a supply network from this synthetic communication network and calculate the ESRI. We calculate Spearman’s rank correlation coefficient, $\rho$, between the ESRI as calculated on the full, real HSN and on the reconstructed subgraph. After repeating these steps for 100 times with $m=1/3$, $p(c|s) = 0.21$ and $p(c|\lnot s) = 9.3 \times 10^{-5}$, we find an average Spearman correlation of $\langle \rho (ESRI_{HSN}, ESRI_{reconstr})\rangle = 0.563(6)$. In SI Text 7 we address the shortcomings mentioned above one by one and discuss the expected magnitude of the introduced errors. We find that the most relevant effect is caused by the limited market share with a drop of correlation of $\Delta \langle \rho \rangle = 0.31$, followed by the limited overlap, adding another, $\Delta \langle \rho \rangle = 0.13$. The effects from network reconstruction reduce the correlation by only $\Delta \langle \rho \rangle = 0.0004$, which is remarkably small. We calculate the probability that a node that is among the 0.1% riskiest nodes of the subsample is also among the riskiest 0.1% of *all* nodes and find 32.9(82)%. The probability that one of the top 0.1% of the subsample nodes is among the top 1% of the full network is 47.7(99)%.

## Discussion

We show that mobile phone metadata can be used to reasonably reconstruct the flow of goods between firms in an economy, i.e., the entire supply network of a nation. The reconstruction is possible because of the similarity of the communication- and the supply layer of the inter-firm network. This method is one of the very few alternatives to obtain a comprehensive view on national supply networks, when there is no VAT or payment system data available.

Based on the reconstructed supply network we calculate economic systemic risk and find that a small core of about 65 high systemic risk firms have the potential to affect large parts of the economic activity. Apart from these core firms, systemic risk of companies is generally small. These results agree well with previous results for Hungary, where a core of 32 high systemic risk firms was found to contribute to 45% of the overall systemic risk^18^. With a series of robustness checks we demonstrate the reliability of the results.

Using a large-scale survey on the actual customer-supplier relationships between companies, we find the probability of a supply link to exist, given an existing communication link as $p(s_{ij}|c_{ij}) \approx 0.19$. When thresholding for higher strength of the communication relation $p(s_{ij}|c_{ij})$ the conditional probability increases strongly to 92%. Note that the survey asked for the firms’ *most* critical suppliers. It is almost certain that in the FCN we observe connections to suppliers that are perhaps important but were not classified as critical in the survey, causing $p(s_{ij}|c_{ij})$ to be underestimated. Landline phones are still common practice in many firms; these communication links are not covered, thus further underestimating the overlap of communication and supply links.

We find that the degree exponents of the reconstructed supply network (RSN), $\alpha _k^{RSN} \approx 2.18$, and the exact Hungarian supply network (HSN), $\alpha _k^{HSN} \approx 2.40$, are similar; the degree exponent of the human-human communication network (HCN) is much larger, $\alpha _k^{CN} \approx 4.89$. Also for the average nearest neighbor degree and the local clustering coefficient the topology of the RSN is more similar to the topology of the exact HSN than to the HCN.

We showed that the FCN and the HSN are most similar when thresholding communication strength to $d_{ij}>30\mathrm {s/d}$. We sample supplier directions using external information on companies’ industry sectors and from input-output tables. Link weights are estimated by the product of firm sizes. Future improvement of the reconstruction method could be reached by using additional information contained in the FCN, such as asymmetries in the calling behavior, temporal patterns in the sequence of calls, as well as using dependencies of supply link weights on communication intensity.

Because the reconstruction process is stochastic, we calculate an ensemble of systemic risk profiles and investigate the inter-quartile ranges. For high- and low-systemic risk firms the inter-quartile ranges are small, indicating that results are stable. However, for firms of intermediate systemic risk the inter-quartile ranges are relatively large, indicating that risk changes strongly with the direction of one or a few links. This agrees well with previous results for the Hungarian supply network, where approximately a third of the riskiest firms were found to constitute the periphery of the high systemic risk core. These firms ‘inherit’ the high systemic risk status from important firms by being critical suppliers to these firms^18^. Also in banking networks it is well known that individual links may dramatically increase systemic risk^11,29^. Although the ESRI correlates strongly with firm size, the high systemic risk core is not predicted well by firm size.

The method has several limitations. We systematically investigate the error introduced by the imperfect overlap of the communication- and supply layers, the limited market share of the mobile phone provider, and the reconstruction of the link directions. In a simulation study we find an average rank correlation between the true ESRI in the HSN and the ESRI on a carefully simulated synthetic firm communication network of $\langle \rho (ESRI_{HSN}, ESRI_{reconstr}) \rangle = 0.563$. The limited market coverage and the imperfect link overlaps contribute most to this effect. We expect $\langle \rho (ESRI_{HSN}, ESRI_{reconstr}) \rangle$ to be higher in reality since it is based on the estimate for *p*(*s*|*c*) that is a lower bound. Further, despite the limited correlation, our method allows us to capture heterogeneity in shock spreading well and uncovers the localized effects of up- and downstream cascades on the firm level that traditional methods such as input-output models cannot describe.

There are four limitations that could not be addressed explicitly. First, firms use many more communication channels than mobile phones, such as landlines, e-mail or physical mail, and a growing number of new interaction channels, such as social media or online portals. Nevertheless, we assume that, if the supply relation is sufficiently strong, firms become more and more likely to use mobile phones to arrange spontaneous meetings, inform partners about delays, coordinate the quality, quantity and timing of deliveries, fix dates, provide support, etc.

Second, due to the anonymity of the telecommunication data it is not possible to perform targeted surveys on the customers of the phone provider. To reach significant overlap of the survey respondents and the customers of the phone provider, untargeted surveys need a response rate of a considerable fraction of firms within a country.

Third, another consequence of the anonymity of the data is that –by definition– firms cannot be identified and concrete policy statements can only be made on the level of the network or firms' industry sector membership. However, within the anonymity constraints, the effect of heterogeneous shocks in relation to economic sectors and geography can still be investigated. This is important since recent work has shown that heterogeneity in the initial economic shocks can cause dramatically different economic outcomes^17,45^.

Fourth, we can’t quantify to what extent the COVID-19 pandemic has influenced the presented results. Already in the early days of the pandemic, supply chain disruptions due to the lockdowns in China were reported and discussed widely. In the online survey on which the validation in Fig. 2a is based, around one third of all firms reported supply chain disruptions. However, at the time we collected the mobile phone dataset there was no lockdown, and the economic situation had somewhat relaxed. Nevertheless, if supply chain disruptions and demand reductions due to the pandemic caused changes in the supply network between the survey and the mobile phone data collection period, this would likely underestimate the overlap *p*(*s*|*c*).

Since mobile phone meta data is easily available, the presented method to reconstruct a national production network is cheap, scalable, and easy to implement. It can be used for countries where no tax or survey data is available. The method also captures international links which allow us to identify economic exposures to specific countries. Maybe one of the most interesting features of the method is its temporal resolution—supply relations can be monitored in real-time. This offers the possibility to study how inter firm-links form and rewire on the network-level. Monitoring the restructuring processes of the economy during natural disasters or economic crises are immediate areas of application and could become crucial for monitoring the progress in the green transition, where production networks have to transform such as to no longer produce greenhouse gases.

## Materials and methods

### Data

The anonymized (but fine-grained, device-level) call detail record (CDR) data is mapped to an anonymized ID for each company. The observation period is approximately 125 days in autumn 2020 between two lockdowns. The obtained edge list is aggregated for the whole observation period, grouped by each source/destination anonymized firm ID tuple and the call duration (in seconds) for each arc is summed up. Further, node-level statistics i.e. the number of devices is aggregated. We also calculate a rough location as the centroid of the night-locations of the individual devices. The night-location was previously calculated as described in^46^ for each device.

The firm communication dataset is merged with a commercially available business intelligence database that includes balance sheet information the industry classification in the NACE 2008 system^47^). For details on the anonymization procedure see SI Text 8. For the analysis we drop NACE J61, J62, M70, and N82 to exclude businesses such as call-centers that have telephone activity at the center of their business and would confound the study with exceptionally high numbers of calls.

To compare the reconstructed supply network (RSN) with a real supply network we use a dataset based on granular VAT reporting in Hungary (HSN), described in^18,31^. It contains a link between two firms only if at least two transactions occur in two different quarters. We use the data from 2017, where only transactions with a tax content larger than 1,000,000 Forint (approx. 3000€) are included. Hungarian VAT rates range from a 27% base rate to a 18% and 5% reduced tax rate for certain foods, pharmaceuticals, etc. and a 0% rate for public transport^48^. The calculations presented here are based on an unweighted version of the Hungarian production network.

We further compare the topology of the FCN with a human-to-human communication network (HCN). To this end we use a dataset provided by the same phone provider. It contains CDRs of calls between individual mobile phones which are anonymized with a new key every 24 hours. For this reason we can only analyze the HCN of one day. We choose September 17, 2020, a Thursday during the observation period outside of the holiday season and before the winter lock-downs. On that day we find 144,516 active devices and 154,557 calls.

We use input-output tables containing information on how many intermediate goods or services were used for the overall production of a certain good in a national economy in a given year. We use the input-output table of 2017, it is the latest available of the country studied. It contains 64 sectors in the CPA classification (*Classification of Products by Activity*^49^), which is harmonized with NACE 2008 on level 2.

### Systemic risk

We define the relative output level of firm *i* at time t as $h_i(t) = \frac{x_i(t)}{x_i(0)}$, where *x*(*t*) is firm *i*’s output at time *t*. Let an initial firm *i* default by setting $h_i(0)= h^\text{d}_i(0)= h^\text{u}_i(0) =0$. Subsequently the shock from firm *i*’s default propagates *downstream* along the out-links by updating all other firms’ output according to their production function

2 $$\begin{aligned} x_l^{\text {d}}(t+1) = f_l\Big (\sum _{j=1}^{n} W_{jl} h_j^\text {d}(t) \delta _{p_j,1}, \dots , \sum _{j=1}^{n} W_{jl}h_j^\text {d}(t) \delta _{p_j,m}\Big ) \quad , \end{aligned}$$

where $\delta _{ij}$ denotes Kronecker’s delta, and *upstream* along the in-links by updating

3 $$\begin{aligned} x_l^{\text {u}}(t+1)= & {} \sum _{j=1}^{n} W_{lj}h_j^\text {u}(t) \quad . \end{aligned}$$

At time *T*, the algorithm has converged and we define the vector $h_j(T)=\min (h_j^d(T),h_j^u(T))$ to calculate the economic systemic risk index as

4 $$\begin{aligned} \mathrm{ESRI}_{i} = \sum _{j=1}^{n} \frac{s_j }{\sum _{l=1}^n s_l }\big (1-h_j(T) \big ) \quad , \end{aligned}$$

where $s_i$ denotes the size of firm *i*. For more details on the algorithm and the definition of the production function, see SI Text 8.

## Supplementary Information

Supplementary Information.

## Data Availability

The mobile phone data is highly sensitive and cannot be shared. The survey data has been collected under GDPR restrictions and can also not be made publicly available.
