# Supplementary material for: Monitoring supply networks from mobile phone data for estimating the systemic risk of an economy
Source: Sci Rep. 2022 Aug 3;12:13347. doi: 10.1038/s41598-022-13104-5 (PMC9349293; doi:10.1038/s41598-022-13104-5)
Supplement: Supplementary file 1 — Supplementary Information. [file 41598_2022_13104_MOESM1_ESM.pdf]

Supplementary Information for “Monitoring supply networks from mobile phone data for estimating the systemic risk of an economy”

Tobias Reisch[1,2,*], **Georg Heiler**[2,3,*], **Christian Diem**[2,4], **Peter Klimek**[1,2], **and Stefan Thurner**[1,2,5,+]

[1]Section for Science of Complex Systems, Center for Medical Statistics, Informatics and Intelligent Systems, Medical University of Vienna, A-1090 Vienna, Austria
[2]Complexity Science Hub Vienna, A-1080 Vienna, Austria
[3]Institute of Information Systems Engineering, TU Wien, A-1040 Vienna, Austria
[4]Institute for Finance, Banking and Insurance, Vienna University of Economics and Business, A-1020 Vienna, Austria
[5]Santa Fe Institute, Santa Fe, NM 85701, USA
[*]T.R. and G.H. contributed equally.
[+]Corresponding author, e-mail: stefan.thurner@meduniwien.ac.at

SI Text 1: Firm sample description

To protect the anonymity of the data providing company and the investigated firms, we cannot disclose the exact number of firms in our firm communication dataset. Here, we compare the composition of our sample with respect to number of firms per sector and aggregate turnover per sector with official numbers retrieved from the national statistics office.

We calculate the share of firms, n_s, in a sector, s, as $n_s = N_s/N$, where N_s is the number of firms in s and N is the total number of firms. Supplementary Figure S1a plots the firm share per sector as reported by the statistics office, n_s^{official}, against the share in our sample, n_s^{sample}. The two quantities are highly correlated, as can be confirmed by Pearson's correlation coefficient $r = 0.84$ ($p < 10^{-17}$) and a Spearman rank correlation of $\rho = 0.88$ ($p < 10^{-21}$). We repeat the procedure for the aggregate turnovers, T_s, for each sector, s, with T denoting the total turnover and $t_s = T_s/T$ the turnover share. In SI Fig. S1 we plot the turnover share reported by the statistics office, t_s^{official}, against the turnover share in our sample, t_s^{sample}. The quantities are still highly correlated, albeit less than the number of firms per sector, with a Pearson correlation of $r = 0.74$ ($p < 10^{-11}$) and a Spearman rank correlation of $\rho = 0.75$ ($p < 10^{-12}$).

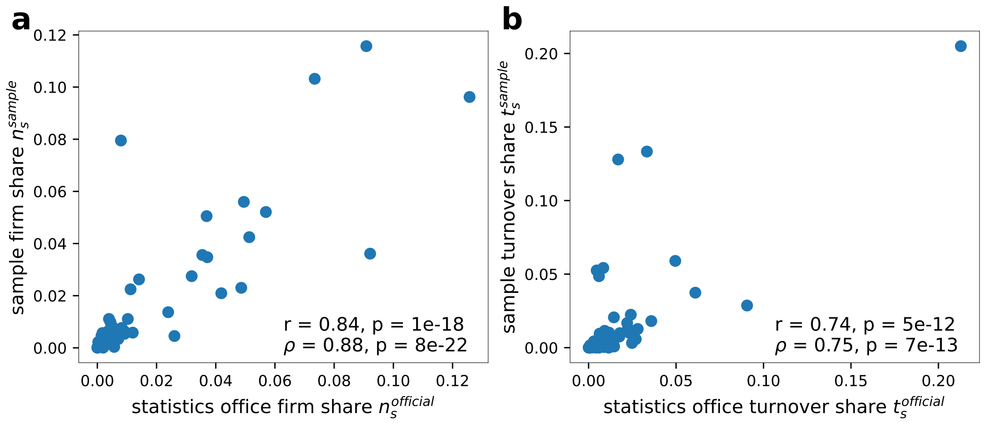

Figure S1. Comparison of the sample composition and official data. (a) Relative number of firms per sector n_s as published by the statistics office, n_s^{official}, plotted against the same value in our dataset, n_s^{sample}. We denote Pearson's correlation coefficient with r and Spearman's rank corrleation with ρ. (b) Share of turnover per sector t_s as published by the statistics office, t_s^{official}, plotted against the share of turnover in our dataset, t_s^{sample}. Both the number of firms and the aggregate turnover per sector of our sample correlate strongly with official numbers published by the statistics office.

SI Text 2: Calculating the conditional probabilities $p(s|c)$ and $p(c|s)$

To calculate conditional probabilities describing the overlap of the communication and supply layer, we use data from a survey conducted in the country of the FCN that was conducted between April 8 and 20, 2020. The survey asked questions on the general business results of the past half year, the outlook for the near future and how the firms expected to be affected by the COVID-19 crisis in the near future. Additionally, the survey contained a part asking for the ten most critical suppliers and ten most critical customers. From this survey we construct a supply network, that we can compare with the FCN. The survey was conducted in cooperation with a large business representation organization, that has very high coverage and represents firms across all sectors except agriculture. The business representation organization sent survey invitation links to all its members and provided the online-tool for the survey as well. The survey was sent out to 102,386 companies; more than 5,955 firms replied to the supply network part, declaring more than 17,393 customer-supplier relations. To keep the privacy of the companies, the data is co-anonymized. This means that the metadata is made available to all parties prior to the data collection process and, subsequently, only anonymized data is made available to the researchers.

We quantify the overlap to find a link x_{ij}, $x \in \{c,s\}$, in one layer when a link y_{ij}, $y \in \{s,c\}$, in the other layer is present as the conditional probability $p(x|y)$,

$$p(x|y) = \frac{p(x \cap y)}{p(y)}. \tag{1}$$

When comparing the results of the supply chain survey and the firm communication network there is an additional distinction between firms reporting in the survey and firms reported in the survey. Let's denote the buyer-supplier network as the set $\mathscr{S}$ containing all links s_{ij} from reporting firm i to reported firm j. This means

$$i \in \mathscr{R}, \qquad j \in \mathscr{M} \qquad \text{and} \qquad \mathscr{S} = \mathscr{R} \cup \mathscr{M} \quad , \tag{2}$$

where $\mathscr{R}$ is the set of reporting nodes, $\mathscr{M}$ the set of reported (mentioned) nodes and $\mathscr{S}$ the set of all nodes in the network. Please note that the sets $\mathscr{R}$ and $\mathscr{M}$ are not mutually exclusive; a reporting node can also be mentioned by another firm and, hence, be part of both sets.

Let's denote the communication network as the set C of links c_{ij} from firm i to firm j, where $i, j \in \mathscr{C}$, $\mathscr{C}$ being the set of firms in the communication network.

We are interested in the conditional probability of finding a buyer-supplier relationship where there is a communication link. Formally $p(s_{ij}|c_{ij})$. To estimate this, we need to perform a fair comparison and stratify for the fact that only a subset of all buyer-supplier relationships is known. Figure SI Fig. S2 illustrates the problem. The central (black) node has reported in the

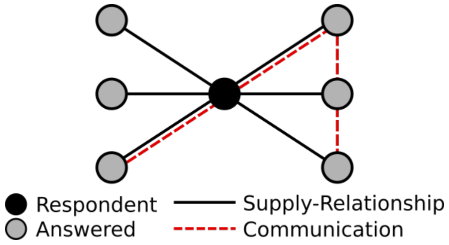

Figure S2. Schematic visualization of how to calculate conditional probabilities using a supply chain survey. By construction only supply links (solid black line) between the reporting (black) and reported (grey) nodes are observable. This means the communication links (broken red lines) need to be limited to links between reporting and reported nodes.

survey and mentioned 6 other nodes (grey). We can only observe links between black and grey nodes, not between two grey nodes, so to perform a fair comparison communication links between two grey nodes need to be excluded from the analysis. We define $\tilde{\mathscr{C}}$ in analogy to the buyer-supplier network as the set of communication links from reporting nodes to mentioned nodes, thereby excluding links between i.e. mentioned nodes

$$\tilde{\mathscr{C}} = \{c_{ij} | i \in \mathscr{R} \wedge j \in \mathscr{M}\}. \tag{3}$$

Now, following Eq. (1) we can write

$$p(s_{ij}|c_{ij}) = \frac{|\tilde{\mathscr{C}} \cap \mathscr{S}|}{|\tilde{\mathscr{C}}|}. \tag{4}$$

To establish indicators for the error of the survey we perform a bootstrap-like simulation. Our simulations corrects for the fact that the distribution of call durations d_{ij} for the subsample in the survey is not representative of the full network. We start by sampling a synthetic supply network based on the empirical communication network using $p(s|c)$ as found in the survey. We use averages of $p(s|c)$ on bins of d_{ij}. Then we draw subsamples of the sample size of the survey ($N \propto 200$). After repeating the process 1500 times, we calculate mean and quartiles and report them in main text Fig. 2b.

For $p(c|s)$ we lack the true distribution of supply link strengths, so we perform a classic bootstrap. For a given conditional probability bin we draw samples of the same size with replacement. We repeat the process 1500 times, calculate mean and quartiles and report them in SI Fig. S3.

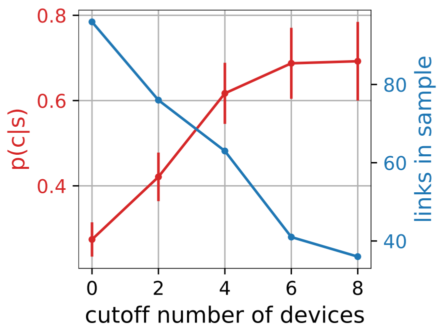

Figure S3. The conditional probability $p(c|s)$ to find a communication link if there is a supply link. We show $p(c|s)$ as a function of the firm size thresholds, which are chosen as proxies for the supply link strength. The blue line shows the number of supply links in the sample used to calculate $p(c|s)$.

SI Text 3: Finding the optimal threshold using Kullback-Leibler divergence

The overlap probabilities $p(s|c)$ and $p(c|s)$ increase when thresholded for call duration and/or firm sizes. Of course on the one hand, when thresholding, the information contained in low-intensity contacts (low call duration, small trade volumes) is lost, while on the other hand, link correlations between the communication and supply layers increase. To balance these two effects, we choose the threshold combination where the topology of the thresholded communication network is most similar to a real production network.

To determine when the topology of the thresholded FCN most resembles a real production network, we calculate the Kullback-Leibler divergence between the thresholded FCN and the HSN,

$$KL = \sum_k p(k^{FCN}) \log\left(\frac{p(k^{FCN})}{p(k^{PNW})}\right). \quad (5)$$

We systematically try threshold combinations for the average call duration per week d_{ij} and the number of devices per firm N_i, (d_{ij}, N_i). As shown in SI Fig. S4a and S4b, the minimal Kullback-Leibler divergence is found for $(30\text{s/d}, 0)$.

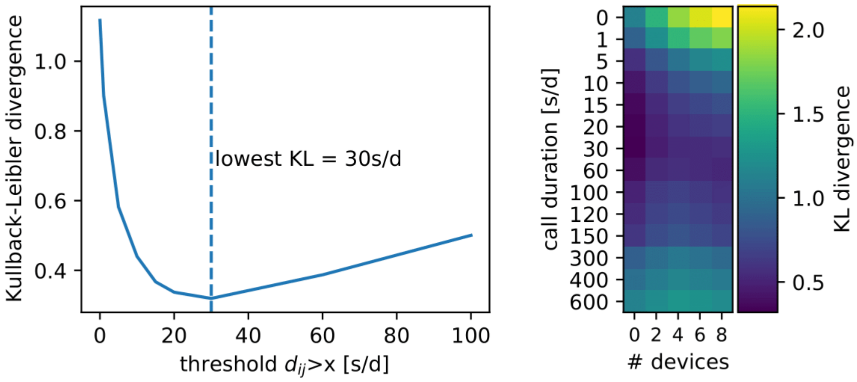

Figure S4. Finding the optimal threshold combination by minimizing the Kullback-Leibler divergence. (a) Kullback-Leibler divergence between $p(k)$ of the HSN and the FCN for different thresholds of the average call duration d_{ij}. The lowest value for KL is at $d_{ij} = 30\text{s/d}$. (b) Heatmap showing KL between the HSN and the FCN for different thresholds of d_{ij} and the number of devices N_i. KL is lowest for $d_{ij} > 30\text{s/d}$ and $N_i > 0$.

SI Text 4 Comparing the network topologies of the FCN, HSN and HCN

Here we compare the network topologies of the FCN, HSN and HCN we discuss the behavior of $p(k)$, $k_{nn}(k)$ and $c(k)$ in greater detail. We begin by characterizing the topology of the firm communication network (FCN). For reference we compare the results to a more directly observed supply network and to a human communication network and show that the topology of the FCN is similar to the known supply network and dissimilar to the social network. Here we describe the network thresholded to only links between firms that have an average interaction duration of more than 30 seconds per day. For the comparison with a real supply network, we compare with the national supply network of Hungary that is obtained through VAT data for 2017[1,2] (henceforth HSN, short for "Hungarian supply network"). The network shows a link if the tax content of the goods exchanged between two firms exceeds 1,000,000 Forint (approx. 3,000 Euro) and if the link occurs in at least two distinct quarters. For details on the supply network data, see Materials and Methods and[1]. To investigate the difference between the inter-firm communication network and a social communication network (HCN) between humans we use a dataset on calls of individual devices by the same mobile phone provider. The data is for one day during the studied period because the IDs of individual devices are re-anonymized daily, preventing us from studying the communication network for longer than 24h. For details on the HCN we refer to Materials and Methods.

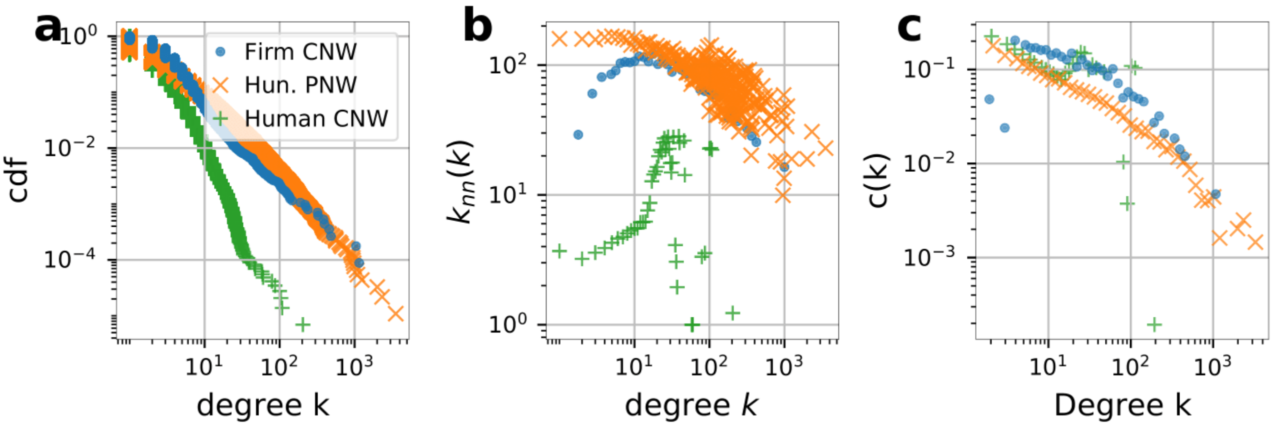

Figure S5. Similarity of the communication and supply layer of the inter-firm multilayer network. (a) Counter cumulative distribution function $p(k > x)$ for the degree k of the FCN (blue dots), HSN (orange x's) and HCN (green pluses). (b) Average nearest neighbor degree $k_{nn}(k)$ and (c) local clustering coefficient c(k) for the three networks. Especially the large-k behavior of the FCN and the HSN is very similar compared to the HCN.

In SI Fig. S5a we show the degree distribution $p(k)$ of the three networks. In complex networks the degree distribution is often skewed to the right with a fat tail, corresponding to the fact that a few hubs are interacting with many nodes, while the majority of nodes interacts with only a few nodes. The FCN has an average degree of $\langle k^{FCN} \rangle = 4.79$. Its degree distribution has a maximum at $k^{FCN} = 2$ and has a fat tail that can asymptotically be described by a power-law

$$p(k) \propto k^{-\alpha}, \tag{6}$$

which we fit with a maximum likelihood estimator using Python's *powerlaw* package[3]. We find $\alpha_k^{FCN} = 2.18(12)$ for $k^{FCN} > 30$. The PNW does not show the increase for small k but also exhibits a fat tail that can be well fitted by the power law from Eq. 6 with $\alpha_k^{HSN} = 2.40(3)$ for values of $k^{HSN} > 30$. The average degree is $\langle k^{HSN} \rangle = 2.1$. For the Japanese production network an average degree of $\langle k^{JPN} \rangle = 8.0$ and degree exponents of $\alpha_k^{JPN,in} = 2.35$ and $\alpha_k^{JPN,out} = 2.26$ have been reported[4].

For the HCN we find an average degree of $\langle k^{HCN} \rangle = 4.75$. There the decrease of $p(k)$ for high values is stronger, with a degree exponent of $\alpha_k^{HCN} = 4.89(26)$. Our results are between the exponents found in two studies in the literature. For a statistically validated communication network in Shanghai, Li et al. fit exponentially truncated power laws and report $\alpha_k^{in} = 2.76$ and $\alpha_k^{out} = 2.90$ for the in- and out-degree, respectively[5]. Onnela et al. report for a mobile phone communication network in Spain, where they find a tail exponent of $\alpha_k = 8.4$[6]. The difference can be perhaps explained by a change in the way mobile phones are used, as, Li et al. argue, a claim which is also supported by a significantly lower degree exponent of $\alpha_k = 2.1$ for landlines, as Onnella et al. report.

The mixing patterns of a network have a strong influence on its structure and function. If high-degree nodes tend to interact with other high-degree nodes, the network is called assortative, if high-degree nodes are more likely to interact with low-degree nodes, the network is called disassortative. For supply networks disassortative mixing has been reported[4]; for social networks we expect assortative mixing[5,6]. To investigate the degree-degree correlations we plot the average nearest neighbor degree

$$\langle k_{nn} \rangle = (1/k_i) \sum_{j \in \mathcal{N}_i} k_j, \tag{7}$$

where $\mathcal{N}_i$ is the set of neighbor nodes of i. In SI Fig. S5b we plot $\langle k_{nn} \rangle$ as a function of k for the FCN (orange), PNW (blue) and HCN (green). For the FCN $\langle k_{nn} \rangle(k)$ shows an increase for small values below $k < 10$ and then shows decreasing trend for larger k. For very small values the network is assortative, for intermediate and large k the firm communication network is disassortative. For the PNW we find $\langle k_{nn} \rangle$ to be relatively flat for small $k < 10$ and then decrease for large k, thereby showing that the PNW is disassortative for $k > 10$. This result agrees well with previous studies on the Japanese supply network, which was also shown to be disassortative. The HCN increases to values around $k \approx 30$ and then decreases quickly, suggesting the existence of two regimes; a low-degree regime, where assortative mixing patterns dominate, and a high-degree regime, where disassortative mixing is predominant. For the mobile phone communication network of Shanghai very similar results were found. There the authors associate the assortative mixing for nodes with 'reasonable degree' to the social network of calls and the disassortative mixing of high-degree nodes with hotlines or robots[5]. This suggests, that for 'human' callers, the network is assortative.

The local cohesiveness around one node is typically measured by the local clustering coefficient c_i. It is defined as the number of closed triangles of node i with its neighbors, t_i, divided by the number of possible triangles

$$c_i = \frac{2t_i}{k_i(k_i - 1)}. \tag{8}$$

The average local clustering coefficients $\langle c \rangle$, along with their expected value for a random network p is shown in SI Tab. S1. All clustering coefficients are comparably small, but still large compared to random networks. Supplementary Figure S5c shows average c_i as a function of degree $c(k)$. For the FCN (orange) and the HSN (blue) $c(k)$ shows a very similar decay. The HCN (green) does not show such a decay, but a decrease for values below $k \approx 20$ and an increase for values between $k \approx 20$ and $k \approx 40$. This weak, peaked dependence of c on k for a mobile phone network was also reported in[5].

Table S1. Network characteristics for four networks in our study. We show the average degree $\langle k \rangle$, the average clustering coefficient $\langle c \rangle$ the linking probability for a random network of the same density p and the average nearest neighbor degree $\langle k_{nn} \rangle$ for the unthresholded FCN, for the RSN, the HCN and the HSN.

Network	$\langle k \rangle$	$\langle c \rangle$	p	$\langle k_{nn} \rangle$
FCN (no threshold)	23.4	0.24	0.0024	516
RSN ($d_{ij} > 30$s/d)	4.79	0.09	0.0006	77
HCN	2.1	0.09	0.000015	3.7
SNW	4.75	0.06	0.000052	157

SI Text 5: Calculating Economic Systemic Risk

Here we describe how the ESRI is calculated. We keep our notation closely to the one of[2].

Given a supply network W, where W_{ij} describes the value of products, of type p_i, delivered from firm i to firm j. The vector p with element $p_i \in \{1, 2, \ldots, m\}$ indicates the product type produced by firm i. We identify the product p_i with a firm's industry affiliation. The amount of input k firm j uses is $\Pi_{jk} = \sum_{i=1}^{n} W_{ij} \delta_{p_i,k}$, here δij denotes Kronecker's delta. A firm's production function is an increasing function $f_i(\Pi_{i1}, \ldots, \Pi_{im})$ that describes how much firm i can produce with a given set of inputs $\Pi_{i1}, \ldots, \Pi_{im}$. Conversely it also allows to assess how much production drops if the amount Π_{ik} of input type k is reduced.

The generalized Leontief production function is a generalization of the regular Leontief production function — with functional form $x_i = \min\left(\frac{1}{\alpha_{i1}} \Pi_{i1}, \frac{1}{\alpha_{i2}} \Pi_{i2}\right)$ — and a linear production function — with functional form $x_i = \frac{1}{\alpha_{i1}} \Pi_{i1} + \frac{1}{\alpha_{i2}} \Pi_{i2}$ — and is defined as

$$x_i = \min\left[\min_{k \in \mathscr{I}_i^{\text{es}}} \left(\frac{1}{\alpha_{ik}} \sum_{j=1}^{n} W_{ji} \delta_{p_j,k}\right), \beta_i + \frac{1}{\alpha_i} \sum_{k \in \mathscr{I}_i^{\text{ne}}} \sum_{j=1}^{n} W_{ji} \delta_{p_j,k}\right] \tag{9}$$

where the set, $\mathscr{I}_i^{\text{es}}$, denotes all input types k, that are deemed essential for production of firm i and thus entering the production in a Leontief way, the set $\mathscr{I}_i^{\text{ne}}$ denotes all input types k that enter the production of firm i in a linear way. In our study, firms in sectors up to NACE category F43 are assumed to have a physical production process that is sensitive to the lack of physical inputs. We denote $\mathscr{I}_i^{\text{es}} = \{\text{A01, ..., F43}\}$ and $\mathscr{I}_i^{\text{ne}} = \{\text{G45, ..., U99}\}$ for all $i \in \{\text{A01, ..., F43}\}$. Firms with NACE categories larger than F43, such as retail trade, wholesale trade or services are assumed to have only non-essential inputs, $\mathscr{I}_i^{\text{ne}} = \{\text{A01, ..., U99}\}$ for all $i \in \{\text{G45, ..., U99}\}$. The parameter $\alpha_{ik} = \frac{\sum_{j=1}^{n} W_{ji} \delta_{p_j,k}}{\sum_{l=1}^{n} W_{il}}$ is the fraction of firm i's output that it spends on the input type k, the parameter $\alpha_i = \frac{\sum_{j=1}^{n} W_{ji}}{\sum_{l=1}^{n} W_{il}}$ is the overall fraction of output that is spend on all inputs and β_i is another parameter inferred from the supply network and defined as the attainable production level if only essential inputs $k \in \mathscr{I}_i^{\text{es}}$ are available, i.e.,

$$\beta_i = \left(\sum_{l=1}^{n} W_{il}\right) \frac{\sum_{k \in \mathscr{I}_i^{\text{es}}} \sum_{j=1}^{n} W_{ji} \delta_{p_j,k}}{\sum_{j=1}^{n} W_{ji}} \quad . \tag{10}$$

We list the necessary equations to compute the ESRI. First, the following objects have to be defined. The downstream impact matrix Λ^{d} defined by

$$\Lambda_{ji}^{\text{d}} = \begin{cases} \Lambda_{ji}^{d1} & \text{if } p_j \in \mathscr{I}_i^{\text{es}} \\ \Lambda_{ji}^{d2} & \text{if } p_j \in \mathscr{I}_i^{\text{ne}} \end{cases} \quad , \tag{11}$$

and the elements of Λ^{d1} and Λ^{d2} are defined as

$$\Lambda_{ji}^{d1} = \begin{cases} \frac{W_{ji}}{\sum_{\iota=1} W_{\iota i} \delta_{p_\iota, p_j}} & \text{if } W_{ji} > 0 \quad , \\ 0 & \text{else} \end{cases} \quad , \tag{12}$$

$$\Lambda_{ji}^{d2} = \begin{cases} \frac{W_{ji}}{\sum_{l=1} W_{li}} & \text{if } W_{ji} > 0 \quad , \\ 0 & \text{else} \end{cases} \quad . \tag{13}$$

Similarly, the upstream impact matrix is defined by

$$\Lambda^{\mathrm{u}}_{ji} = \begin{cases} \frac{W_{ij}}{\sum_{l=1}^{n} W_{il}} & \text{if} \quad W_{ij} > 0 \quad , \\ 0 & \text{else} \quad , \end{cases} \tag{14}$$

Second, to calculate the ESRI of firm i the initial exogenous shock parameter is chosen to be $\psi_i = 0$ and $\psi_j = 1$ for the other firms $j \neq i$.

Third, the following equations are iteratively computed to update the downstream and upstream impeded production levels of firms at time point t. We define the relative output level of firm i at time t as $h_i(t) = \frac{x_i(t)}{x_i(0)}$.

1. Compute the dynamic intraindustry market share for each firm j

$$\sigma_j(t) = \min\left(\frac{s_j^{\mathrm{out}}(0)}{\sum_{l=1}^{n} s_l^{\mathrm{out}}(0) h_l^d(t) \delta_{p_l,p_j}}, 1 \right) \quad . \tag{15}$$

2. Compute, for essential inputs $k \in \mathscr{I}_i^{\mathrm{es}}$ of firm i the fraction available as

$$\tilde{\Pi}_{ik}(t) = 1 - \sum_{j=1}^{n} \sigma_j(t) \Lambda^d_{ji} \left(1 - h_j^{\mathrm{d}}(t)\right) \delta_{p_j,k} \quad . \tag{16}$$

3. Compute, for non-essential inputs, $k \in \mathscr{I}_i^{\mathrm{ne}}$, of firm i the fraction available as

$$\tilde{\Pi}_{ik'}(t) = 1 - \sum_{k \in \mathscr{I}_i^{\mathrm{ne}}} \sum_{j=1}^{n} \sigma_j(t) \Lambda^d_{ji} \left(1 - h_j^{\mathrm{d}}(t)\right) \delta_{p_j,k} \quad . \tag{17}$$

4. Update for each firm i the relative production level reduced by downstream shocks

$$h_i^{\mathrm{d}}(t+1) = \min\left[\min_{k \in \mathscr{I}_i^{\mathrm{es}}} \left(\tilde{\Pi}_{ik}(t) \right), \tilde{\Pi}_{ik'}(t), \psi_i \right] \quad , \tag{18}$$

5. Update for each firm i the relative production level reduced by upstream shocks

$$h_i^u(t+1) = \min\left[\sum_{j=1}^{n} \Lambda^{\mathrm{u}}_{ji} h_j^{\mathrm{u}}(t), \psi_i \right] \quad . \tag{19}$$

The iteration continues until the algorithm reaches a stable state at time

$$T \equiv \min_t \{ t \in \mathbb{N} | \max\left(h^d(t) - h^d(t+1), h^u(t) - h^u(t+1) \right) \leq \varepsilon \} + 1 \quad , \tag{20}$$

with $\varepsilon = 10^{-2}$ as convergence threshold.

Then the ESRI_i of firm i is computed as

$$\mathrm{ESRI}_i = \sum_{j=1}^{n} \frac{s_j}{\sum_{l=1}^{n} s_l} \left(1 - h_j(T)\right) \quad , \tag{21}$$

where s_i denotes the size of firm i. The quantity can be interpreted as the fraction of production in the network that is (temporarily) impeded if firm i fails (temporarily).

For details of the derivation see[2] Appendix G.

Note that the calculation is computationally intensive and scales badly, because with growing network size the number of ESRI_i to calculate grows linearly and the convergence times grows by 2 times matrix multiplication costs. For this reason we only show the 1000 most risky firms in Figure 3 (d).

SI Text 6: Extended analysis of the ESRI profiles

We are not only interested in the median damage a company can do, but also in the scenario where the damage is largest. In SI Fig. S6 we plot the maximal ESRI per node of 100 reconstructed supply networks. Reconstructing 100 supply networks and calculating ESRI yields a distribution for every node. Supplementary Figure S6 shows the maximum of each distribution. Note that the maxima are not all from the same configuration. The maximal damage is $max(ESRI) = 0.53$ and the high systemic risk core consists of around 100 firms, with the majority of nodes having an ESRI around $ESRI \approx 0.25$.

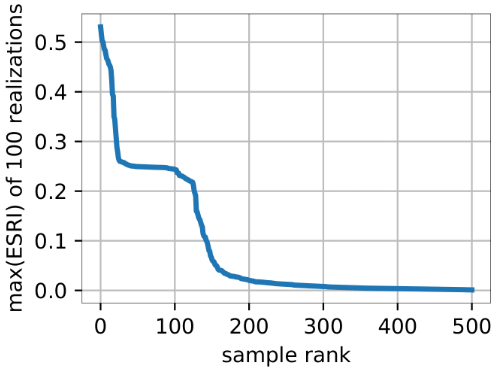

Figure S6. Maximal worst-case economic systemic risk. We plot the maximal ESRI per node found after simulating 100 configurations of the RSNW ordered from highest to lowest. The shown values are the maximal damage a node does in 100 scenarios, they do not occur all together in one configuration.

Table S2. NACE lvl. 2 classification of the firms in the high systemic risk plateau.

code	sector name	frequency	percent
C28	Manufacture of machinery and equipment n.e.c.	10	15.4%
C17	Manufacture of paper and paper products	6	9.2%
D35	Electricity, gas, steam and air conditioning supply	5	7.7%
C16	Manufacture of wood and of products of wood and cork,...	5	7.7%
C23	Manufacture of other non-metallic mineral products	4	6.2%
C10	Manufacture of food products	4	6.2%
C22	Manufacture of rubber and plastic products	4	6.2%
C25	Manufacture of fabricated metal products, except machinery and equipment	4	6.2%
K64	Financial service activities, except insurance and pension funding	4	6.2%
C11	Manufacture of beverages	2	3.1%
C24	Manufacture of basic metals	2	3.1%
C20	Manufacture of chemicals and chemical products	2	3.1%
C29	Manufacture of motor vehicles, trailers and semi-trailers	2	3.1%
C32	Other manufacturing	2	3.1%
F43	Specialised construction activities	2	3.1%
A01	Crop and animal production, hunting and related service activities	1	1.5%
G47	Retail trade, except of motor vehicles and motorcycles	1	1.5%
E38	Waste collection, treatment and disposal activities; materials recovery	1	1.5%
C33	Repair and installation of machinery and equipment	1	1.5%
C31	Manufacture of furniture	1	1.5%
F42	Civil engineering	1	1.5%
C21	Manufacture of basic pharmaceutical products and pharmaceutical preparations	1	1.5%

SI Text 7: Limitations for systemic risk calculation

Our study is subject to several limitations, in particular (i) the error due to faulty direction/weight estimation, (ii) the limited market coverage of the phone provider (resulting in limited agreement even if $p(s|c) = p(c|s) = 1$) and (iii) the imperfect overlap of the two networks limiting the possible accuracy. In the following, we address all of these shortcomings one by one and discuss the size of the introduced biases and errors. We end with a simulation to estimate the error introduced by all shortcomings combined.

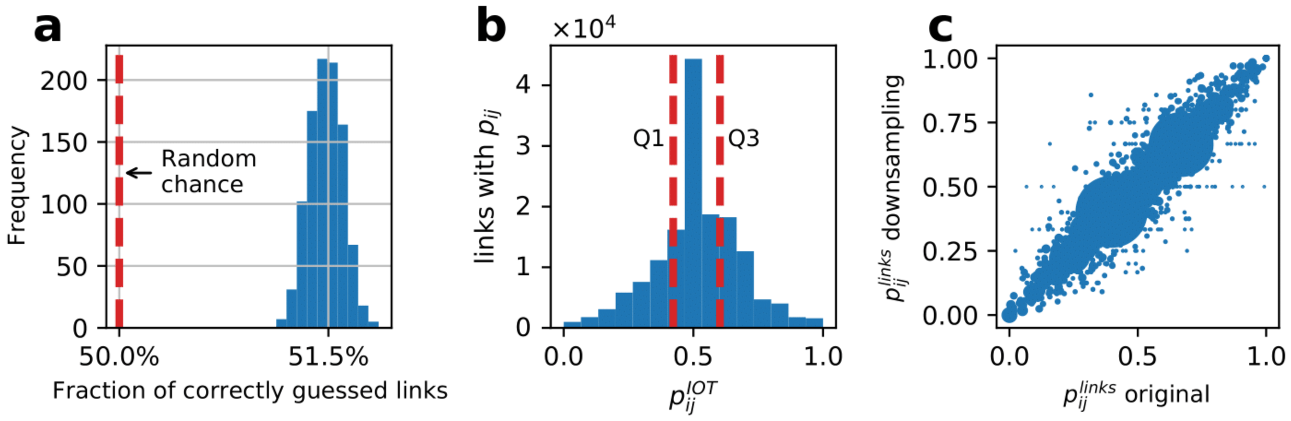

Figure S7. Accuracy of the link direction estimation. (a) We aggregate the weights of the HSN to a NACE lvl. 2 input-output table. From this we compute p_{ij} and estimate the link direction for every link. We calculate the fraction of correctly guessed link directions and show the result of 1000 iterations in the blue histogram. The average fraction of correctly guessed links is 51.5%. Using no additional information from input-output tables, i.e. assuming $p_{ij} = 0.5$ would result in an average fraction of correctly guessed links of 50% (red vertical line). (b) Number of links associated with a given value of the direction probability p_{ij}^{IOT}. The distribution is centered around 50%, indicating that for the majority of the links there is no clear flow direction. The red vertical lines indicate the first and third quartile at 42% and 60%, respectively. The peak at 50% is because many trade links are inside one sector, resulting in $p_{ii} = 0.5$. (c) Fraction of links p_{ij} from i to j in the original network and after downsampling the links from the aggregated network for one reconstruction. Although, as shown in panel (a), the individual directions are not captured very well, on the aggregate level the fraction of links pointing from i to j after reconstruction correlates strongly with the values from the original network. After 1000 iterations we find an average Pearson correlation of $\langle r \rangle = 0.91(1)$.

We study the error introduced by sampling the link directions by simulating the reconstruction process on a real supply network topology and then validating it by comparison with the true network. We use the Hungarian supply network and start by aggregating the trade volumes to an input-output table. Then we remove the direction information from the network and sample new link directions according to the probabilities obtained from the input-output table and Eq. (1). We compare the true and simulated link directions and calculate which fraction was guessed correctly. Supplementary Figure S7a shows results of repeating this experiment 1000 times. The mean overlap is 51.5% with a standard deviation of 0.1%. This result, although significantly better than what would be expected from random chance (red line in SI Fig. S7b), is surprisingly low. It can be explained by investigating the probabilities associated with the links in Hungary. If most links were between sectors with a direction as polarized as the relationship between agriculture and the food industry, more links would be guessed correctly. However, as shown in SI Fig. S7b the majority of links has probabilities between 42% and 60% (the lower and upper quartile, shown as red lines). Nevertheless, even though many direct links are guessed incorrectly, on the sector level the proportion of links from sector i to sector j $p_{ij} = L_{ij}/(L_{ij} + L_{ji})$ are captured well. We find an average Pearson correlation of the true, empirical sector wise link directions p_{ij}^{emp} and the simulated sector wise link directions p_{ij}^{sim} of $\langle r(p_{ij}^{emp}, p_{ij}^{sim}) \rangle = 0.91(1)$, see also SI Fig. S7c.

As in most countries, there is more than one mobile phone provider in the country where the mobile phone data is from. This results in a market share m less than one. As is schematically shown in SI Fig. S8a, this leads to a large fraction of links that are not accounted for, since we only consider calls between companies who are customers of the mobile phone provider. The graph containing only the links between a set of nodes in a network is called the *induced subgraph* of the respective set of nodes. To quantify the error introduced by limited coverage we use the real supply network of Hungary and compare the systemic risk as calculated on the full network with the systemic risk calculated on an induced subgraph. We investigate the effect of a market coverage of $m = 1/4$, $m = 1/3$ and $m = 1/2$ using the following steps.

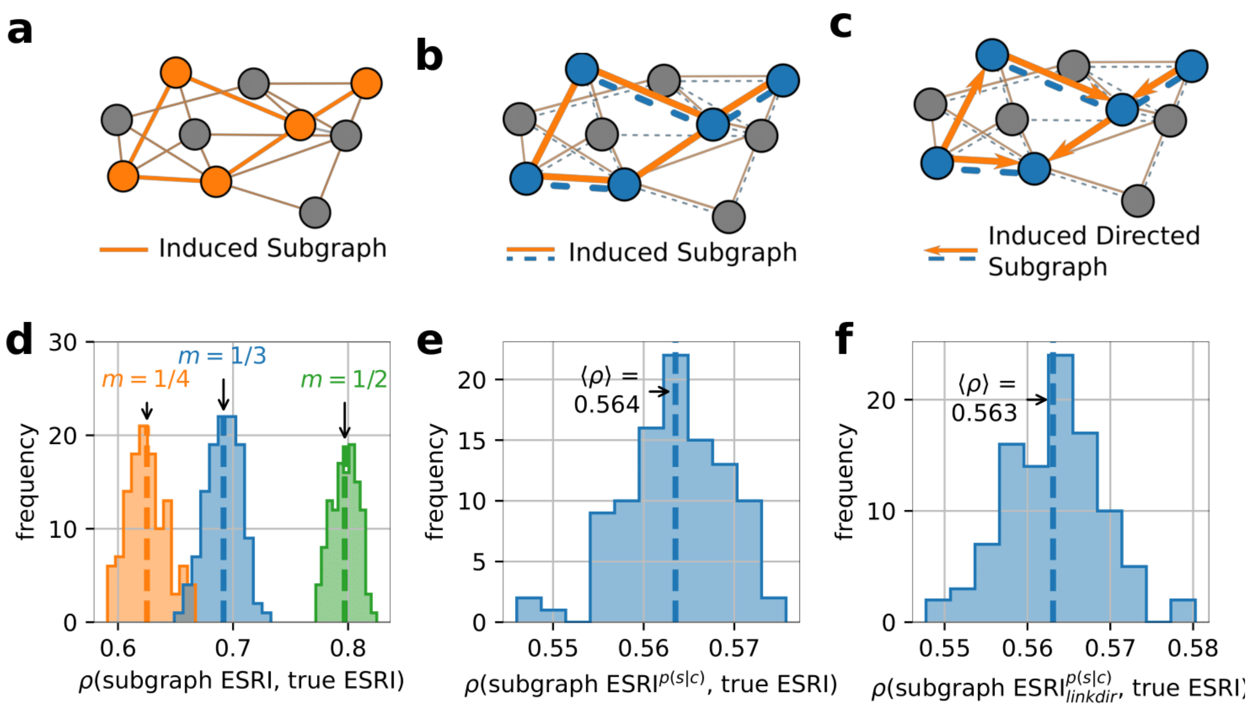

Figure S8. Limitations for systemic risk calculations. Perform simulations on the HSN to estimate the size of the error introduced by considering (a) the induced subgraph of calls between customers of a mobile phone company with market share m, (b) the induced subgraph on a multilayer network with imperfect overlap and (c) the induced subgraph on a multilayer network with imperfect overlap where the link directions need to be estimated. (d) The correlation coefficient of the ESRI calculated on a full network and a subgraph of size $m = 1/3$ (blue) and $m = 1/2$ (green). The histograms show the results of 120 iterations and the vertical links highlight the median values $\langle\rho\rangle_{m=1/4} = 0.63(2)$, $\langle\rho\rangle_{m=1/3} = 0.69(1)$ and $\langle\rho\rangle_{m=1/2} = 0.80(1)$. (e) On top of the induced subgraph we limit the overlap of the communication and supply layer by generating a synthetic communication layer using $p(s|c) = 0.21$. The correlation coefficients of 100 simulations with $m = 1/3$ are shown in the blue histogram, the mean correlation coefficient is $\langle\rho\rangle = 0.564(5)$ (red vertical line). (f) As an additional step we add the reconstruction of the link directions as described in the main text. The correlation coefficients of 100 simulations are shown in the blue histogram, the mean correlation coefficient is $\langle\rho\rangle = 0.563(6)$ (red vertical line). Arguably the largest contribution to the overall error is caused by the limited market share, followed by limited overlap $p(c|s) < 1$. The error introduced due to the link direction estimation is only marginal.

1. Calculate ESRI for the full Hungarian network.
2. Draw a sample of nodes according to market share m, calculate the induced subgraph.
3. Calculate ESRI on induced subgraph.
4. Correlate ESRI of induced subgraph with “true" ESRI of these nodes.
5. Repeat from 2. and calculate the histogram of correlation coefficients.

Supplementary Figure S8d shows the results of 120 iterations, the average Spearman correlation coefficient $\rho(ESRI_{full}, ESRI_{subgraph})$ for $m = 1/4$ (orange) is $\langle\rho\rangle_{m=1/4} = 0.63(2)$, $m = 1/3$ (blue) is $\langle\rho\rangle_{m=1/3} = 0.69(1)$ and $m = 1/3$ (green) is $\langle\rho\rangle_{m=1/2} = 0.80(1)$.

The next step is to quantify the error introduced by the imperfect link correlations, $p(s|c) < 1$ and $p(c|s) < 1$. Supplementary Figure S8b illustrates the imperfect overlaps of the communication (broken blue lines) and the supply (solid orange lines) layers. We generate a fake communication network, based on the HSN and the probabilities to find a communication link where a supply link is present $p(c|s)$ and where no supply link is present $p(c|\neg s)$. It is not possible to measure $p(c|\neg s)$ directly.

Nevertheless we can compute it from known quantities

$$p(c|\neg s) = \frac{p(c) - p(c|s)p(s)}{1 - p(s)}. \tag{22}$$

We are interested in the effect on top of the error introduced by the incomplete market coverage, therefore we sample nodes according to a market share of $m = 1/3$ and calculate the induced subgraph. To isolate the effect, however, we keep the directions from the HSN and investigate their effect in the next step. The modified algorithm works as follows:

1. Calculate ESRI for the full Hungarian network.
2. Generate "fake" mobile phone network for Hun using $p(c|s)$ and $p(c|\neg s)$
3. Calculate ESRI on the simulated mobile phone network.
4. Correlate ESRI of the simulated network with the "true" ESRI.
5. Repeat from 2. and make histogram of correlation coefficients.

Supplementary Figure S8e shows the results of 100 iterations assuming $m = 1/3$ and $p(c|s) = 0.21$. The mean Pearson correlation is $\langle\rho\rangle_{p(s|c)} = 0.564(5)$, demonstrating a substantial shift of $\Delta\langle\rho\rangle = 0.13$ compared to the result not including $p(s|c) < 1$.

Finally, we study the combined effect of the limitations described above, as shown in SI Fig. S8c we calculate the effects of a limited market share, imperfect link correlations and an inaccurate link direction estimation. We use the empirical network topology of Hungary, simulate a mobile phone network and then estimate the link directions. The process follows the steps below.

1. Calculate ESRI for the full Hungarian network.
2. Generate“fake" mobile phone network for Hun using $p(c|s)$ and $p(c|\neg s)$
3. Draw a sample of nodes according to market share m, calculate induced subgraph.
4. Reconstruct the directions using input-output tables.
5. Calculate ESRI on induced subgraph of the simulated phone network.
6. Correlate ESRI of induced subgraph with "true" ESRI of these nodes.
7. Repeat from 2. and make histogram of correlation coefficients.

Supplementary Figure S8f shows the results for 100 iterations with $m = 1/3$ and $p(c|s) = 0.21$. We find an average Pearson's correlation coefficient of $\langle\rho(ESRI_{full}, ESRI_{reconstr})\rangle = 0.563(6)$. Compared to the previous simulation the estimation of the link directions adds only a small error $\delta\langle r\rangle = 0.0004$ to the final result.

SI Text 8: Anonymization procedure

The firm communication dataset is merged with a commercially available business intelligence database that was made available to the mobile phone provider. This database includes balance sheet information from which we proxy the firm's sizes by their total assets, and on their industry classification in the NACE 2008 system[7]). Because this information would potentially make it possible to identify individual firms, the merged data does not leave the premises of the phone company. All calculations were executed there and then anonymized. In this form the data was handed to us and was destroyed at the phone company after the project.

To calculate conditional probabilities describing the overlap of the communication and supply layer, we perform a large survey to obtain ground truth data, for details see SI Text 2. To keep the privacy of the firms, the data is co-anonymized. This means that metadata is exchanged between the researchers and the mobile phone provider and a shared anonymization procedure is employed. Finally, only fully anonymized data is made available to the researchers.

References

1. Borsos, A., Stancsics, M. *et al.* Unfolding the hidden structure of the hungarian multi-layer firm network. Tech. Rep., Magyar Nemzeti Bank (Central Bank of Hungary) (2020).

2. Diem, C., Borsos, A., Reisch, T., Kertész, J. & Thurner, S. Quantifying firm-level economic systemic risk from nation-wide supply networks (2021). *arXiv preprint arXiv:2104.07260*.

3. Alstott, J., Bullmore, E. & Plenz, D. powerlaw: a Python package for analysis of heavy-tailed distributions. *PloS one* **9**, e85777 (2014).

4. Fujiwara, Y. & Aoyama, H. Large-scale structure of a nation-wide production network. *The Eur. Phys. J. B* **77**, 565–580 (2010).

5. Li, M.-X. *et al.* A comparative analysis of the statistical properties of large mobile phone calling networks. *Sci. Reports* **4**, 1–12 (2014).

6. Onnela, J.-P. *et al.* Analysis of a large-scale weighted network of one-to-one human communication. *New J. Phys.* **9**, 179 (2007).

7. Regulation (EC) No 1893/2006 of the European Parliament and of the Council of 20 December 2006 establishing the statistical classification of economic activities NACE Revision 2 and amending Council Regulation (EEC) No 3037/90 as well as certain EC Regulations on specific statistical domains Text with EEA relevance (2006). http://data.europa.eu/eli/reg/2006/1893/oj, retrieved 20th august 2021.
